# Histological and molecular insights in to in vitro regeneration pattern of *Xanthosoma sagittifolium*

**DOI:** 10.1038/s41598-023-33064-8

**Published:** 2023-04-10

**Authors:** Sangita Bansal, Manoj K. Sharma, Shivangi Singh, Parampara Joshi, Pooja Pathania, Era V. Malhotra, S. Rajkumar, Pragati Misra

**Affiliations:** 1 Tissue Culture and Cryopreservation Unit, ICAR- National Bureau of Plant Genetic Resources (NBPGR), New Delhi, 110012 India; 2 Division of Genomic Resources, ICAR- National Bureau of Plant Genetic Resources (NBPGR), New Delhi, India; 3 Sam Higgimbottom University of Agriculture and Technology, Prayagraj, UP India

**Keywords:** Biotechnology, Plant sciences

## Abstract

A study on the effect of various phytohormonal combinations on in vitro propagation of Cocoyam [*Xanthosoma sagittifolium* (L.) Schott] was conducted to develop an improved and efficient in vitro regeneration protocol for its mass multiplication. Histological analysis to understand the in vitro regeneration pattern and genetic fidelity assessment of regenerated plants were also carried out. Single shoots excised from in vitro established cultures of *X. sagittifolium* were used as explants. Among the 32 different phytohormonal combinations tested, indirect organogenesis with intervening callus phase was observed on majority of the media combinations. Meristematic clump formation was optimally achieved on all the tested media combinations with maximum 43.54 ± 0.51 shoot primordia on MS medium containing 0.2 mg/L BAP + 0.1 mg/L NAA followed by 36.44 ± 0.76 shoot primordia on MS medium having 2.5 mg/L TDZ. Micro-morphological analysis of different morphogenetic structures revealed that the regeneration of cocoyam is well executed via meristematic nodules, shoot primordia formation that may evolve in to proper shoots. Adventitious shoots (> 2 cm) were successfully (100.00 ± 0.00%) rooted on the half-strength MS medium containing IBA (0.05–1.0 mg/L) and IAA (0.05–0.5 mg/L). The number of roots ranged from 0.78 ± 0.31 on the control half-strength MS medium to 13.94 ± 0.46 on half-strength MS supplemented with 1.0 mg/L IBA. Considering somaclonal variations as a potential restriction to in vitro multiplication of plants, genetic stability was assessed using 40 ISSR primers. The PCR amplification profiles obtained from all the tested propagules (calli, meristematic clumps, regenerated plantlets) were similar to the mother plants indicating the homogeneity of the individuals raised through the regeneration protocol reported here.

## Introduction

Cocoyam [*Xanthosoma sagittifolium* (L.) Schott] is a monocotyledonous, herbaceous, perennial and vegetatively propagated plant of the family Araceae. It is commonly known as “Arrow elephant ear”, “cocoyam” or “Tania” which produces edible, starch-rich underground corms. It originated from tropical America and is cultivated in some countries such as Nigeria, Ghana, Cameroon, India, etc. in Asia and Africa as a subsistence food crop^1^. Cocoyam is rich in vitamin B6, copper, carbohydrates, iron and potassium. It is one of the plants with the lowest allergy potential. Consuming cocoyam can increase energy, maintain sugar levels, promote and maintain intestinal health and prevent high blood pressure. In some countries like Africa, it is also used as a remedy against burns^2^. Its life cycle starts with shoot emergence and ends with corm formation and includes three growth phases; (i) the first phase which lasts for about 5 months, contains initial 2 months of slow growth. In this phase, sprouting and maximum leaf development take place in the presence of rainfall; (ii) the second phase of around 2 months involves vigorous shoot formation. The plant attains maximum height in this phase; (iii) the third and last phase embraces the development of corms and cormels under dry conditions^3^. Overall, mature plants can produce a large amount of foliage in the first 6–9 months, and produce up to 10 or more corms within 10 months^4^.

Preserving cocoyam under field conditions is risky because diseases or natural catastrophes can cause the loss of genetic resources. Cocoyam is propagated through its corms that enhance the risk of pathogen distribution. The major diseases of *Xanthosoma* are the Dasheen mosaic caused by the virus and cocoyam root rot disease (CRRD) caused by *Pythium myriotylum*. The pathogen can affect the plant at different growth stages; early infection leads to stunting and dieback of plants whereas late infection causes chlorosis and reduced yields. The disease can result in yield loss of up to 100% under conducive environmental conditions^5,6^. In vitro propagation technique is a possible solution to the major problems of vegetatively propagated plants associated with pathogen dissemination and subsequent loss of vigour and productivity^7^, health and quality^8,9^. The ideal concentration and combination of plant growth regulators (PGRs) required for in vitro propagation are different from species to species, genotype to genotype and explant source. The composition of PGRs needs to be optimized accurately to achieve an effective rate of multiplication for a genotype^10^. In micropropagation, it is very important to establish the hormonal balance for each phase of the developmental process^9^.

Limited studies are available on in vitro shoot multiplication, somatic embryogenesis, and tuberization of cocoyam using Murashige and Skoog medium supplemented with various hormone combinations including benzylaminopurine (BAP), kinetin, thidiazuron (TDZ), 2,4-dichlorophenoxyacetic acid (2,4-D), indole-3- butyric acid (IBA), and 1-naphthalene acetic acid (NAA)^11,12^. Further none of these studies analysed the histological changes and genetic stability of developing propagules during in vitro propagation. The present study reports an improved in vitro propagation protocol through meristematic nodule formation in the species for the first time. Histological evaluation to decipher the regeneration pattern of the in vitro culture system and molecular study to ascertain the genetic integrity of regenerated plantlets are also done. Available literature shows that the major limiting factors in any micropropagation protocol are the rate of shoot multiplication and genetic uniformity of regenerated plants. Therefore, the present study was undertaken to analyze the effect of different hormone combinations on *Xanthosoma sagittifolium* multiplication, understand its in vitro regeneration pattern through micro-morphological evaluation of different morphogenetic structures and devise an optimum protocol for producing genetically uniform planting material in large numbers. This is the first study on the micro-morphological evaluation of in vitro multiplication of *X. sagittifolium* along with molecular analysis for assessment of the genetic integrity of regenerants.

## Results and discussion

**Figure 1:**
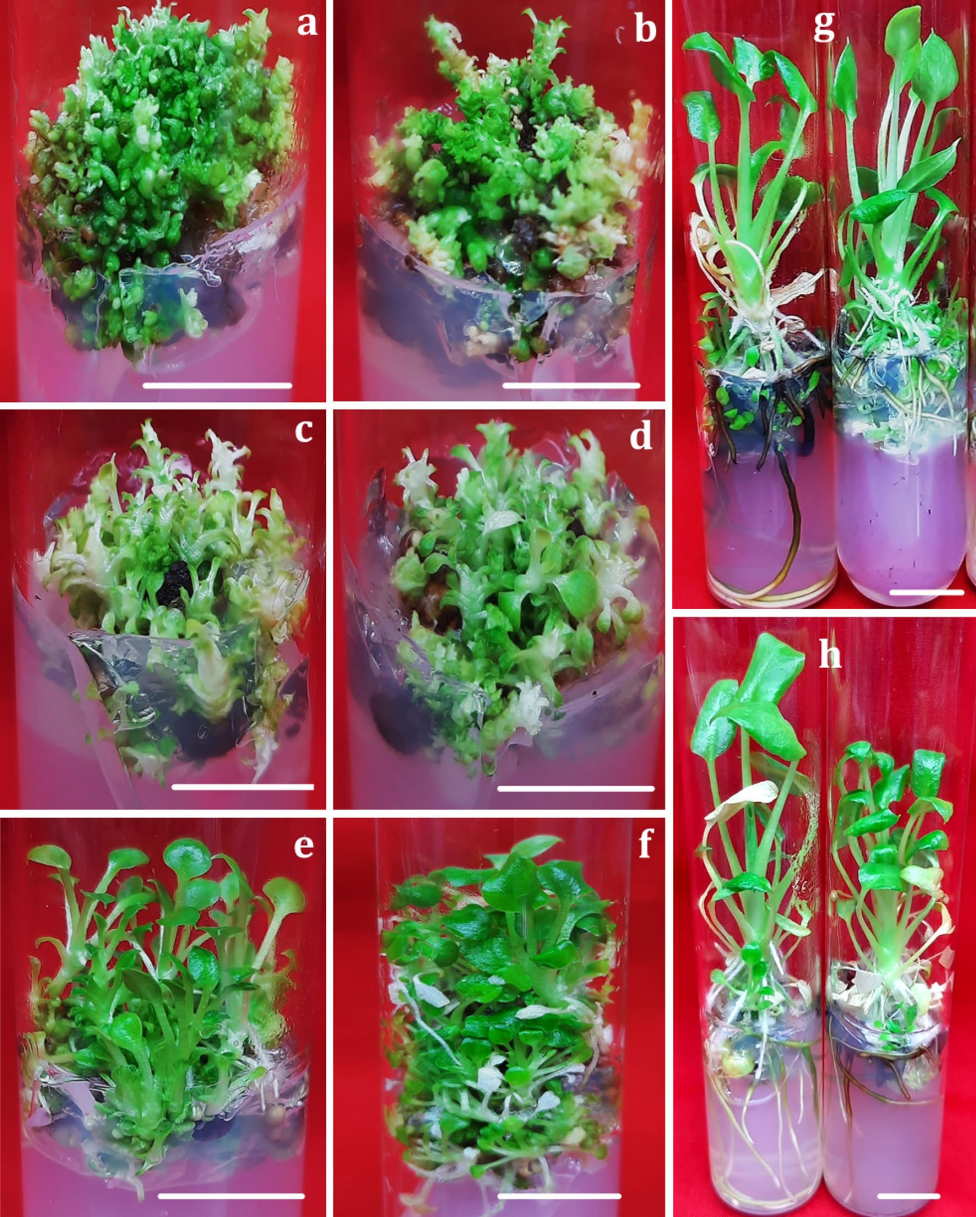
In vitro multiplication response of *Xanthosoma sagittifolium* (Scale bar = 1.25 cm), where (**a**, **b**): meristematic clumps obtained on MS + 0.2 mg/L BAP + 0.1 mg/L NAA and MS + 2.5 mg/L TDZ; (**c**, **d**): meristematic clumps/ shoots obtained on MS + 2.0 or 2.5 mg/L BAP; (**e**–**f**): shoots multiplied on MS + 0.5 mg/L BAP + 0.01 or 0.05 mg/L NAA; (**g**–**h**): plantlets elongated on MS + 0.5 mg/L BAP + or 0.01 or 0.05 mg/L NAA.

**Figure 2:**
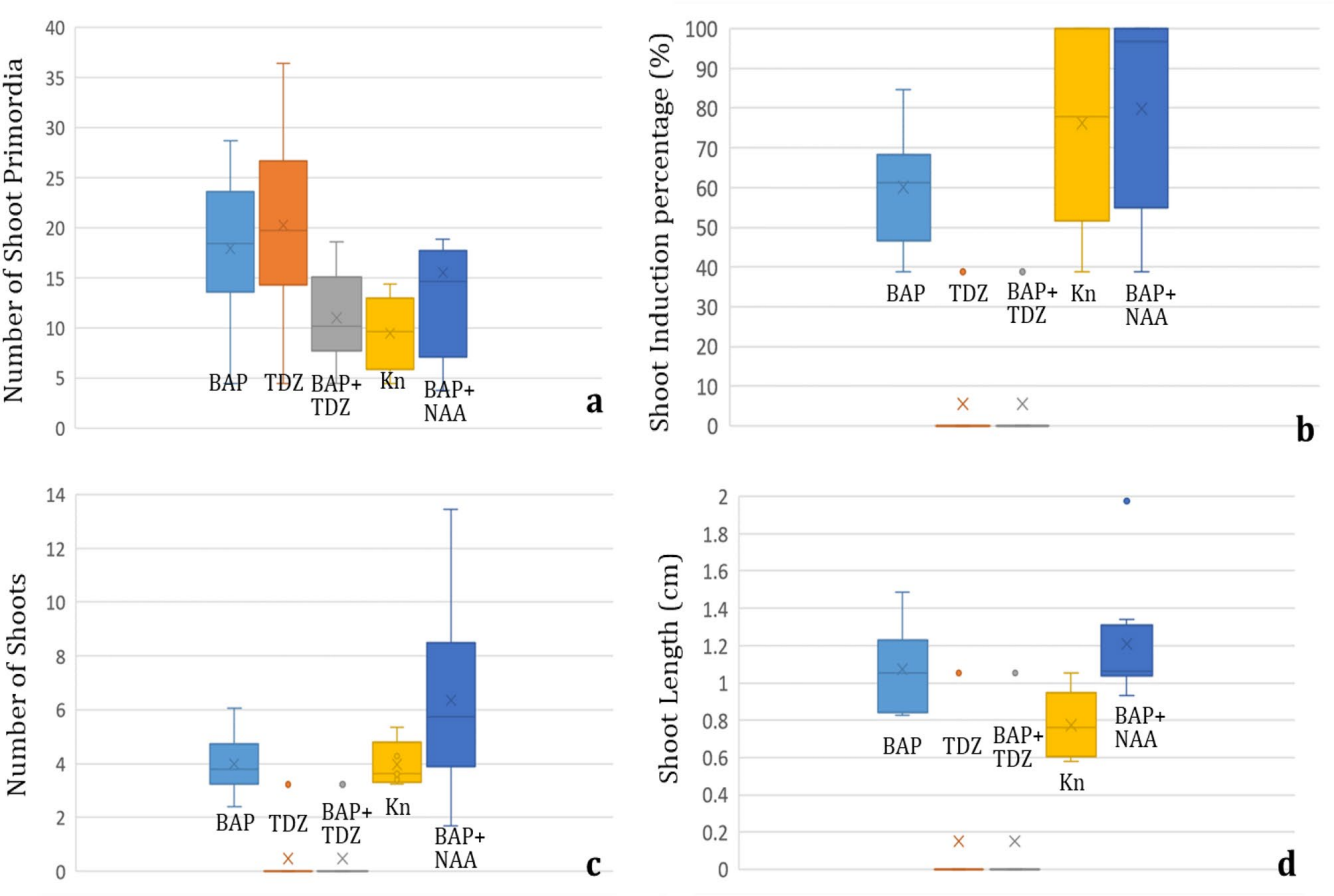
Box plots representing the effect of different media combinations on; (**a**) number of shoot primordia per meristematic clump, (**b**) shoot induction percentage, (**c**) number of shoots per explant and (**d**) shoot length in cm.

Direct organogenesis was not achieved, and callusing was observed on majority of the media combinations except on basal MS medium and MS supplemented with either 0.5 mg/L BAP or 0.2 or 0.5 mg/L kinetin. Different cytokinins resulted in varied regeneration responses of either meristematic clump formation or shoot formation (Fig. 1). Explants inoculated on different culture media combinations started to swell within 2 weeks, while the meristematic clumps were induced after 6 weeks. Meristematic clumps were formed on all the tested media combinations with maximum of 43.54 ± 0.51 shoot primordia induced on MS medium containing 0.2 mg/L BAP + 0.1 mg/L NAA followed by 36.44 ± 0.76 shoot primordia on MS medium having 2.5 mg/L TDZ (Table 1, Fig. 2a). Different cytokinins responded differently with respect to meristematic clump formation with TDZ having highest median value (around 20) for the number of meristematic nodule or shoot primordia followed by BAP combination (Fig. 1a). A previous study by Wada et al.^12^ reported direct organogenesis with multiplication rate of 4.5–4.83 shoots/ explant on MS media with 2.5 mg/L BAP and 0.5 mg/L NAA.

**Table 1:** Effects of different phytohormone combinations on in vitro multiplication of *Xanthosoma sagittifolium.*

S. no	Culture media composition (Basal MS + phytohormones)	Concentration (mg/L)	Meristematic clumps (Mean ± SE)	Shoot induction % (Mean ± SE)	Number of shoots (Mean ± SE)	Shoot length in cm (Mean ± SE)
	Control (Basal MS)	–	4.44 ± 0.34^a^	38.69 ± 1.82^b^ (38.46)	3.22 ± 0.24^c^	1.05 ± 0.15^efgh^
	BAP	0.5	13.61 ± 0.52^gh^	65.45 ± 2.4^ef^ (54.00)	6.06 ± 0.34^jk^	1.48 ± 0.13^j^
		1.0	16.17 ± 0.92^ij^	46.57 ± 1.2^c^ (43.03)	4.72 ± 0.24^ fg^	1.23 ± 0.53^i^
		1.5	18.39 ± 1.35^ k^	55.87 ± 1.27^d^ (48.37)	4.11 ± 0.29^de^	0.84 ± 0.16^def^
		2.0	28.72 ± 0.88^o^	84.74 ± 1.88^ h^ (67.00)	3.78 ± 0.22^ m^	1.06 ± 0.14^efgh^
		2.5	23.57 ± 0.61^hi^	61.27 ± 1.18^e^ (51.51)	3.56 ± 0.20^c^	1.02 ± 0.141^fgh^
		5.0	20.56 ± 0.47d^ef^	68.34 ± 2.12f. (55.76)	2.39 ± 0.28^ef^	0.83 ± 0.13^de^
	TDZ	0.5	26.67 ± 0.77^n^	0.00 ± 0.00^a^ (0.00)	0.00 ± 0.00^a^	0.00 ± 0.00^a^
		1.0	22.39 ± 0.58^ l^	0.00 ± 0.00^a^ (0.00)	0.00 ± 0.00^a^	0.00 ± 0.00^a^
		1.5	17.83 ± 0.84^jk^	0.00 ± 0.00^a^ (0.00)	0.00 ± 0.00^a^	0.00 ± 0.00^a^
		2.0	14.28 ± 0.58^hi^	0.00 ± 0.00^a^ (0.00)	0.00 ± 0.00^a^	0.00 ± 0.00^a^
		2.5	36.44 ± 0.76^p^	0.00 ± 0.00^a^ (0.00)	0.00 ± 0.00^a^	0.00 ± 0.00^a^
		5.0	19.72 ± 0.82^ k^	0.00 ± 0.00^a^ (0.00)	0.00 ± 0.00^a^	0.00 ± 0.00^a^
	BAP + TDZ	1.0 + 0.5	15.11 ± 0.54^j^	0.00 ± 0.00^a^ (0.00)	0.00 ± 0.00^a^	0.00 ± 0.00^a^
		1.0 + 2.0	18.56 ± 0.54^ k^	0.00 ± 0.00^a^ (0.00)	0.00 ± 0.00^a^	0.00 ± 0.00^a^
		2.0 + 1.0	12.33 ± 0.61^ fg^	0.00 ± 0.00^a^ (0.00)	0.00 ± 0.00^a^	0.00 ± 0.00^a^
		1.5 + 2.5	10.17 ± 0.34^cde^	0.00 ± 0.00^a^ (0.00)	0.00 ± 0.00^a^	0.00 ± 0.00^a^
		2.5 + 1.5	8.61 ± 0.58^bc^	0.00 ± 0.00^a^ (0.00)	0.00 ± 0.00^a^	0.00 ± 0.00^a^
		2.5 + 2.5	7.72 ± 0.39^b^	0.00 ± 0.00^a^ (0.00)	0.00 ± 0.00^a^	0.00 ± 0.00^a^
	Kinetin	0.2	11.56 ± 0.46^ef^	77.75 ± 1.854^gh^ (61.85)	4.28 ± 0.23^ef^	0.58 ± 0.06^b^
		0.5	7.28 ± 0.16^b^	100.00 ± 0.00^i^ (90.00)	5.33 ± 0.23^hi^	0.76 ± 0.07^ cd^
		1.2	14.39 ± 0.62^hi^	100.00 ± 0.00^i^ (90.00)	3.61 ± 0.22^ cd^	0.63 ± 0.04^bc^
		2.0	9.61 ± 0.46^ cd^	64.54 ± 1.43^ef^ (53.45)	3.39 ± 0.16^c^	0.84 ± 0.60^def^
	BAP + NAA	0.20 + 0.01	3.78 ± 0.375^a^	51.38 ± 1.23^d^ (45.79)	1.67 ± 0.11^b^	0.93 ± 0.06^defg^
		0.2 + 0.05	15.72 ± 0.619^j^	58.43 ± 1.51d^e^ (49.85)	4.55 ± 0.24^efg^	1.34 ± 0.19i
		0.2 + 0.1	43.54 ± 0.505^q^	73.57 ± 1.76^ g^ (59.06)	5.72 ± 0.23^ij^	1.05 ± 0.19^gh^
		0.5 + 0.01	12.44 ± 0.315 fg	100.00 ± 0.00^i^ (90.00)	9.83 ± 0.29^n^	1.98 ± 0.17^efg^
		0.5 + 0.05	9.78 ± 0.712^ k^	100.00 ± 0.00^i^ (90.00)	13.44 ± 0.26°	1.16 ± 0.18^hi^
		0.5 + 0.1	16.56 ± 0.335^a^	100.00 ± 0.00^i^ (90.00)	6.56 ± 0.18^ k^	1.28 ± 0.12^i^
		0.5 + 0.2	18.83 ± 0.532^ k^	100.00 ± 0.00^i^ (90.00)	4.94 ± 0.19^gh^	1.06 ± 0.18^gh^
		0.1 + 0.15	14.67 ± 0.498^ m^	96.84 ± 1.86^i^ (79.75)	7.17 ± 0.27^ l^	1.03 ± 0.14^fgh^
	BAP + Kinetin	2.5 + 2.5	10.94 ± 0.551d^ef^	0.00 ± 0.00^a^ (0.00)	0.00 ± 0.00^a^	0.00 ± 0.00^a^

Data represents mean ± SE of six replicates per treatment in three repeated experiments. Means within the same column followed the different letters are significantly different according to Duncan’s multiple range test (DMRT) at 5% level.

Arc sine transformation values are given in parentheses.

Earliest shoot induction was witnessed on MS medium with 0.5 mg/L BAP + 0.01 mg/L NAA (within 6 weeks) followed by MS + 0.5 mg/L BAP + 0.05 mg/L NAA (within 8 weeks) and MS + 0.5 mg/L BAP + 0.2 mg/L NAA (within 12 weeks). Shoot induction percentage varied between 38.69 ± 1.82 on basal MS medium to 100% on six media combinations that include MS media supplemented with 0.5 or 1.0 mg/L kinetin and MS media having 0.5 mg/L BAP combined with various concentrations of NAA (0.01–0.2 mg/L). No shoot induction was observed on 12 media combinations containing phytohormone TDZ either alone or in a combination of BAP (Table 1, Fig. 2b). Highest median value of shoot induction percentage was obtained with media combination having BAP + NAA followed by kinetin (Fig. 2b).

Significant variation in shoot number was observed on different media combinations with a minimum of 2.39 ± 0.28 shoots on MS medium with 5.0 mg/L BAP and maximum of 13.44 ± 0.26 shoots on MS + 0.5 mg/L BAP + 0.05 mg/L NAA. No shoots only meristematic clumps were formed on media combinations having TDZ either alone or in combination with BAP (Table 1, Fig. 1b). On media containing BAP and kinetin, the number of shoots ranged between 2.39 ± 0.28 to 6.06 ± 0.34, whereas a significant increase in shoot number was observed on media supplemented with BAP + NAA (Table 1, Fig. 1e, f). In the box plot graph, the highest median value was observed in MS media having BAP + NAA followed by MS media with BAP, indicating a relatively higher shoot number on media containing BAP hormone in comparison with other hormones (Fig. 2c). Shoot length varied between 0.58 ± 0.06 cm on MS medium with 0.2 mg/L kinetin and 1.98 ± 0.18 cm on MS medium having 0.5 mg/L BAP + 0.01 mg/L NAA (Table 1, Fig. 2d). Median values for shoot length for MS media supplemented with BAP or BAP + NAA was found to be almost similar (Fig. 2d) whereas, a significantly lower median was observed for MS media with kinetin.

Medium combination (MS + 0.5 mg/L BAP + 0.05 mg/L NAA) yielding the best multiplication response was tested in 5 other accessions of *X. sagittifolium* (Table 2). Among tested accessions, the number of shoot primordia ranged between 40.78 ± 1.13 to 53.65 ± 1.86 and shoot numbers varied from 10.33 ± 0.82 to 18.46 ± 1.46 with a mean number of 45.41 shoot primordia and 14.79 shoots.

**Table 2:** In vitro multiplication response of five *Xanthosoma* accessions on optimized culture medium (MS** + **0.2 mg/L BAP + 0.1 mg/L NAA**).**

S. no.	Accession name	Number of shoot primordia (Mean ± SE)	Number of shoots (Mean ± SE)	Shoot length (cm) (Mean ± SE)
1	IC0812549	42.865 ± 1.348^b^	16.842 ± 1.142^c^	1.128 ± 0.104^b^
2	IC0582686	40.783 ± 1.134^a^	13.634 ± 1.248^b^	0.644 ± 0.126^a^
3	IC0582687	46.238 ± 1.582^c^	18.464 ± 1.435^d^	1.278 ± 0.145^c^
4	IC0009605	43.524 ± 1.456^b^	14.678 ± 1.375^b^	1.120 ± 0.138^b^
5	IC0582690	53.647 ± 1.864^d^	10.333 ± 0.825^a^	1.040 ± 0.115^b^

Data represents mean ± SE of six replicates per treatment in three repeated experiments. Means within the same column followed the different letters are significantly different according to Duncan’s multiple range test (DMRT) at 5% level.

Based on higher shoot length, four media combinations containing MS with 0.5 mg/L NAA and BAP concentrations from 0.01 to 0.2 mg/L were tested further for shoot elongation. The shoot elongation was best on MS + 0.5 mg/L BAP + 0.01 mg/L NAA with an average shoot length of 4.18 cm (Fig. 1g). This was followed by 3.56 cm shoot length on MS + 0.5 mg/L BAP + 0.05 mg/L NAA, and 2.68 cm on MS + 0.5 mg/L NAA + 0.1 mg/L BAP after 12 weeks of explant inoculation (Fig. 1h).

In vitro propagation has been frequently utilized for the mass multiplication of vegetatively propagated species in less time and space. The success of any micropropagation protocol depends upon the right choice of nutrient media and plant growth hormones. In *Xanthosoma*, nutrient media supplemented with different plant growth regulators (PGRs) including cytokinins BAP, kinetin, and TDZ, used alone or in combination with either each other or auxins IBA, and NAA have been tried to achieve optimal multiplication. Different morphogenic responses have been reported in the previous reports^11,12^. In the present study, the use of different cytokinins has resulted in initial callus induction with subsequent formation of meristematic clumps containing shoot primordia. The highest number of shoot primordia was achieved on media containing 0.2 mg/L BAP + 0.1 mg/L NAA and 2.5 mg/L TDZ. The shoot primordia when cultured singly on media with BAP + NAA produced proper shoots. These results are consistent with the study by Sama et al.^11^, in which the use of 20 μM BAP and 2 μM TDZ in nutrient media (semi-solid or stationary liquid) resulted in higher multiplication rates. Similar to our findings, they described short and stunted shoots on media containing TDZ whereas proper elongated shoots were obtained on BAP. Similarly, Wada et al.^12^ reported the best multiplication response (4.56 and 4.83 shoots in green tannia and purple tannia, respectively) on the MS media having 2.5 mg/L BAP and 0.5 mg/L NAA. Contrary to our results, the exogenous application of BAP on two *Xanthosoma* varieties^13^ resulted in a lower in vitro regeneration response as compared to controls. The best elongation achieved in this study on MS + 0.5 mg/L BAP + 0.01 mg/L NAA with an average shoot length of 4.18 cm is corroborative with the earlier report^12^ with the lengthiest shoot (3.92–4.36 cm) on the MS media + 5.0 mg/L BAP + 1.0 mg/L Kn + 0.5 mg/L NAA. A different study^14^ utilizes a customized liquid bioreactor (temporary immersion culture system) for cocoyam mass multiplication and reported a highest proliferation rate, number of leaves per plant and plant weight on using 20 mg/L sucrose in liquid culture medium.

### Histological analysis

**Figure 3:**
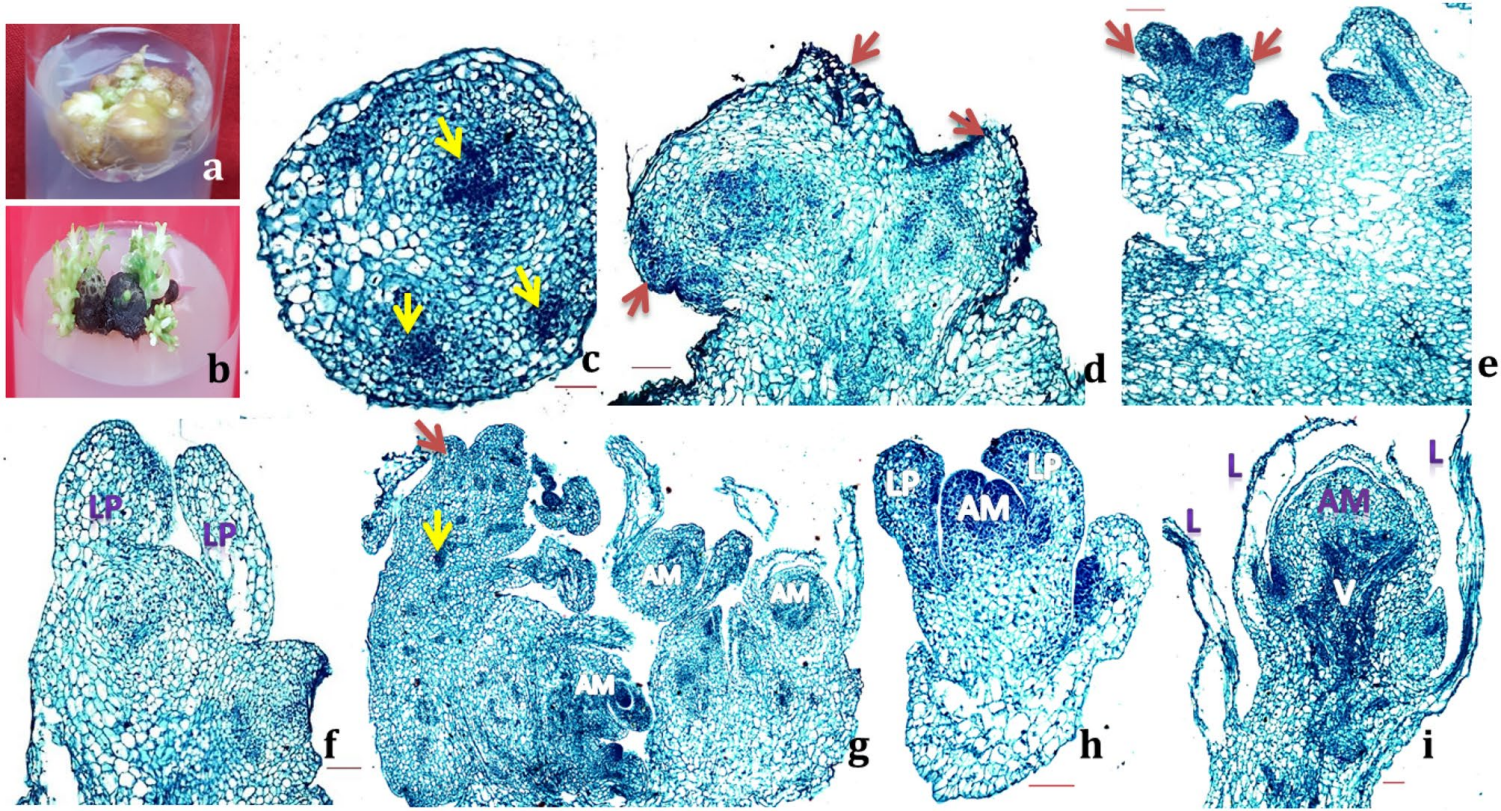
Histological evaluation of in vitro regeneration of *Xanthosoma sagittifolium* (Scale bar = 100 μm); where (**a**). pre-meristematic nodules (**b**). shoot organogenesis (**c**). Meristem mass containing small area of dense, small cells shown by yellow arrows; (**d**–**e**). Pre-meristematic (**d**) and meristematic nodules (**e**) in form of small protrusions at the surface of meristematic clumps shown by red arrows; (**f**). shoot primordia with clear apical dome (AM) and leaf primordia (LP); g. meristematic clump showing different stages of adventitious shoot formation; h. growing meristem to form shoots; (**i**). adventitious shoots containing apical meristem (AM), vascular tissues (V) and leaves (L).

Histological analysis was conducted on various proliferating structures induced during in vitro multiplication to develop a basic understanding of regeneration pattern. Initially a callus-like structure was observed that either produced meristematic clumps through meristematic nodules formation (Fig. 3a) or shoots (Fig. 3b). Micro-morphology of the initial callus-like structure revealed it to contain multiple meristematic zones with compact areas of dense smaller meristematic cells surrounded by larger cells (Fig. 3c). These meristematic zones grew further to give rise to small protrusions on the tissue surface to form pre-meristematic nodule with actively dividing meristematic cells (3d). These pre-meristematic nodules gradually enlarged to produce meristematic nodules or shoot primordia (Fig. 3e, f) exhibiting tunica-corpus organization. Numerous meristematic nodules or shoot primordia containing clear apical meristem surrounded by leaf primordial were visible on the meristematic clumps (Fig. 3g, h). Asynchronous adventitious shoot formation was observed displaying shoot primordia at different developmental stages ranging from early protrusion to well-developed shoot and leaf primordia (Fig. 3g). Shoot primordia differentiated to form organized shoot with several leaves and leaf primordia (Fig. 3g, i). No proper root meristem was observed and all the apical meristems were seen attached with a broad tissue base thus ruling out the possibility of somatic embryogenesis (Fig. 3g).

Our results show that the regeneration of cocoyam is through shoot primordia formation that may develop in to proper shoots. On the majority of cytokinin concentrations (TDZ, kinetin, BAP) used in the present study, an intervening callus phase was observed that initially converted to meristematic nodules and finally formed meristematic clumps containing numerous shoot primordia (supplementary Fig. 1c-e). On medium with BAP, the shoot formation occurs from the shoot primordia and therefore, on these media adventitious shoots, shoot primordia, and meristematic nodule could be observed in different developmental stages suggesting asynchronous development. Whereas, on medium containing, TDZ (alone or with BAP) these shoot primordia failed to convert in to shoots and remained in form of meristematic clumps. No somatic embryogenesis was observed on any of the tested media combinations. Similar to a study on tree peony^15^, the regeneration of *Xanthosoma* involved four distinct phases that included callus with multiple meristematic regions, pre- nodules, meristematic nodules and shoot primordia. Formation of meristematic nodules either directly or indirectly via callogenesis has been reported in a number of species including *Acacia mangium*^16^, *Eucalyptus globulus*^17^, *Humulus lupulus*^18,19^, *Populus euphratica*^20^, *Sclerocarya birrea*^21^ etc. Analogous observations have been made in a study on *Elliottia racemosa*^22^, in which in vitro regeneration from leaf explants took place via shoot organogenesis on a medium containing TDZ and IAA. They have also observed multiple meristematic zones in subepidermal cell layers.

### Rooting and acclimatization

**Figure 4:**
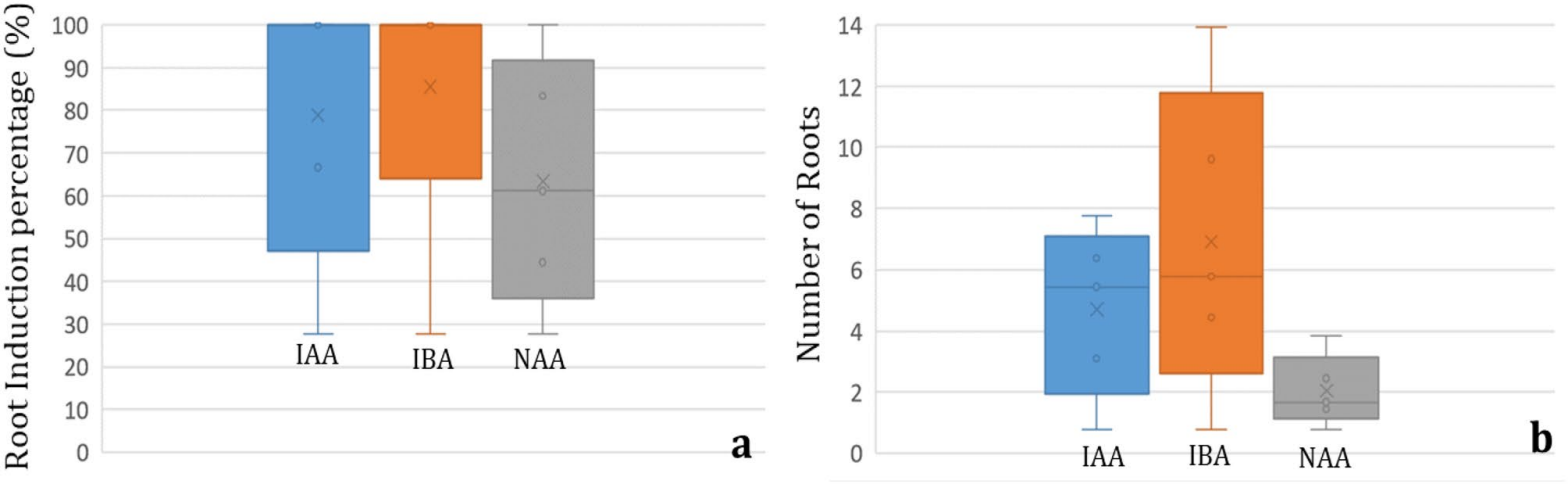
Box plots representing the effect of different media combinations on; (**a**) root induction percentage, (**b**) number of roots per shoot.

Efficient rooting plays an important role in the successful acclimatization of in vitro plantlets. Roots are responsible for the absorption of water and essential nutrients that are required for plant growth and development. For rooting, single shoots (> 2 cm) having 2–3 leaves were placed on different media containing various concentrations of auxins IAA, IBA and NAA. Root induction varied from 27.78 ± 1.86 to 100.00 ± 0.00%. Hundred percent root induction was observed on all the tested concentrations of IBA (0.05–1.0 mg/L) whereas a gradual increase in root induction percentage (44.44 ± 1.2–100.00 ± 0.00%) was obtained with increasing NAA hormone concentration (0.05–1.0 mg/L) with a median value of around 60% (Fig. 4a). Use of IAA resulted in 100.00 ± 0.00% root induction in the concentration range of 0.05–0.5 mg/L and a significant decline (66.67 ± 1.43%) was seen after increasing the concentration to 1.0 mg/L (Table 3, Fig. 4a).

**Table 3:** Effects of different auxins on in vitro rooting response of *Xanthosoma sagittifolium.*

S. no.	Half strength MS + Auxins (mg/L)			Root induction % (Mean ± SE)	Number of roots/ shoot (Mean ± SE)	Root length in cm (Mean ± SE)
	IAA	IBA	NAA			
	0.00	0.00	0.00	27.78 ± 1.86^a^ (31.81)	0.78 ± 0.31^a^	0.82 ± 0.13^a^
	0.05			100.00 ± 0.00^e^ (90.00)	5.44 ± 0.29f.	1.22 ± 0.22^abcd^
	0.10			100.00 ± 0.00^e^ (90.00)	7.77 ± 0.43^ g^	1.71 ± 0.4^de^
	0.50			100.00 ± 0.00^e^ (90.00)	3.11 ± 0.18^ cd^	1.27 ± 0.42^abcd^
	1.00			66.67 ± 1.43^ cd^ (54.74)	6.39 ± 0.28f.	1.53 ± 0.41^ cd^
		0.05		100.00 ± 0.00^e^ (90.00)	4.44 ± 0.15^e^	1.32 ± 0.37^abcd^
		0.10		100.00 ± 0.00^e^ (90.00)	9.61 ± 0.20^ h^	2.13 ± 0.48^e^
		0.50		100.00 ± 0.00^e^ (90.00)	5.78 ± 0.24f.	1.31 ± 0.32^abcd^
		1.00		100.00 ± 0.00^e^ (90.00)	13.94 ± 0.46^i^	5.16 ± 0.82f.
			0.05	44.44 ± 1.21^ab^ (41.81)	1.67 ± 0.58^ab^	1.47 ± 0.44^bcd^
			0.10	61.11 ± 1.18^bc^ (51.42)	1.44 ± 0.29^b^	1.21 ± 0.27^abcd^
			0.50	83.33 ± 2.43^ cd^ (65.90)	2.44 ± 0.45^bc^	1.04 ± 0.28^abc^
			1.00	100.00 ± 0.00^e^ (90.00)	3.83 ± 0.15^de^	0.94 ± 0.22^ab^

Data represents mean ± SE of six replicates per treatment in three repeated experiments. Means within the same column followed the different letters are significantly different according to Duncan’s multiple range test (DMRT) at 5% level.

Arc sine transformation values are given in parentheses.

**Figure 5:**
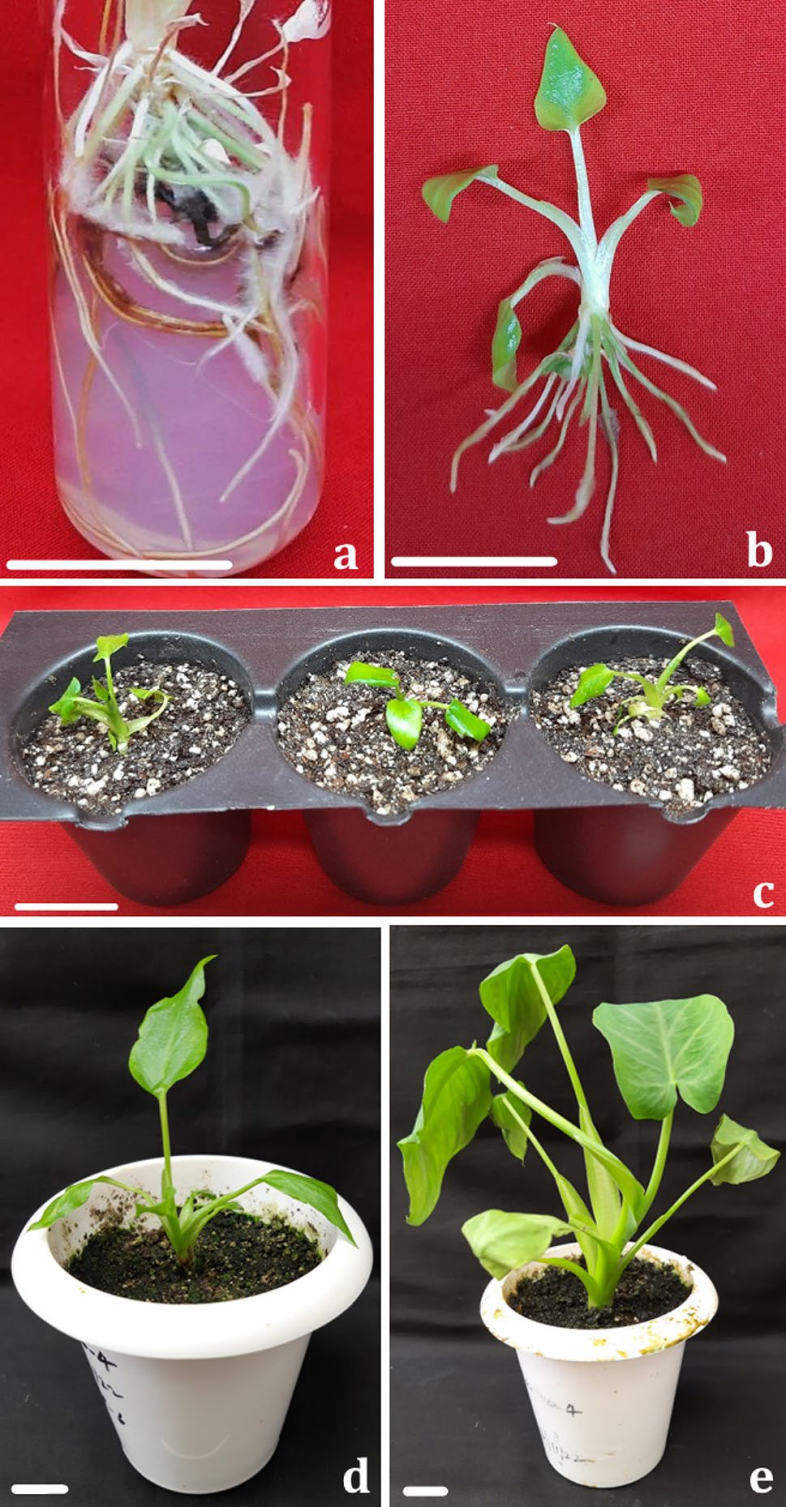
Root induction and acclimatization response of in vitro multiplied shoots (Scale bar = 2 cm), where (**a**, **b**): in vitro rooting, (**c**): 1 week old, hardened plantlet on soilrite, (**d**): 8 weeks old, hardened plantlet, (**e**): 16 weeks old plantlet.

The number of roots varied between a minimum of 0.778 ± 0.31 on basal MS medium to a maximum 13.94 ± 0.46 on MS having 1.0 mg/L IBA (Table 3, Fig. 5a, b). Among tested auxins, NAA was found to be the least responsive (1.44 ± 0.29 to 3.83 ± 0.15 roots) with lowest median value (Fig. 4b) and IBA to be the best (4.44 ± 0.15 to 13.94 ± 0.46 roots) in terms of root numbers (Table 3). With IAA (0.05–1 mg/L), the number of roots ranged between 3.11 ± 0.18 and 7.77 ± 0.43 with a median value of around 5.8 which is similar to the median obtained with IBA concentrations.

Auxins especially IAA, IBA and NAA have been frequently utilized for in vitro adventitious root initiation and development^23^. In vitro rooting process is dependent upon a number of factors that includes genetic factors, culture media composition and endogenous/ exogenous concentration of plant growth hormones^24,25^. Adventitious root formation involves three basic steps including root induction, root initiation and root elongation. A hundred percent root induction achieved in the present report is higher than the previous report in which 93.3% root initiation was observed in green tannia on MS + 2 mg/L IBA and 86.7% initiation in purple tannia on a medium having 2 mg/L NAA. In line with the result obtained in green tannia, IBA was found to be superior to IAA and NAA in terms of root induction in the present report. In context to root numbers, 1 mg/L IBA gave the best response (13.94 roots) which is again higher than the previous report (~ 6 roots) on a medium containing 2 mg/L IBA. As has been reported earlier also, auxin supplementation was found necessary for better and enhanced in vitro rooting in *X. sagittifolium*^7,12,26^.

The rooted plantlets were taken and washed under running tap water to remove any traces of the medium. For hardening, the washed plantlets were planted in pots containing autoclaved soilrite and kept under a controlled atmosphere of mist chamber with 25 ± 1 °C temperature and 75% relative humidity (Fig. 5c). The multiplied plantlets were successfully hardened on soil (Fig. 5d, e) with a survival rate of 83.33% which is better or at par with the *ex vitro* survival rate of green tannia (76.7%) and purple tannia (83.3%) reported earlier^12^.

### Genetic integrity assessment

During in vitro multiplication, higher multiplication pressure due to the application of exogenous plant growth hormones especially synthetic ones tends to induce somaclonal variations. Somaclonal variation is a serious limitation for large scale in vitro clonal propagation of plants. The major causes of somaclonal variations are chromosomal rearrangement, single gene mutations, activation of transposable elements, DNA methylation etc. Molecular markers have been frequently utilized for capturing the genetic variations induced during in vitro propagation or conservation of plants in form of discrete banding patterns. PCR-based single primer amplification reaction (SPAR) methods including random amplified polymorphic DNA (RAPD) and inter simple sequence repeats (ISSR) have been regularly used for genetic fidelity assessment of in vitro propagates^27–31^. The SPAR methods need no prior sequence information, are simple, economic, rapid and require small quantities of DNA^27^.

**Figure 6:**
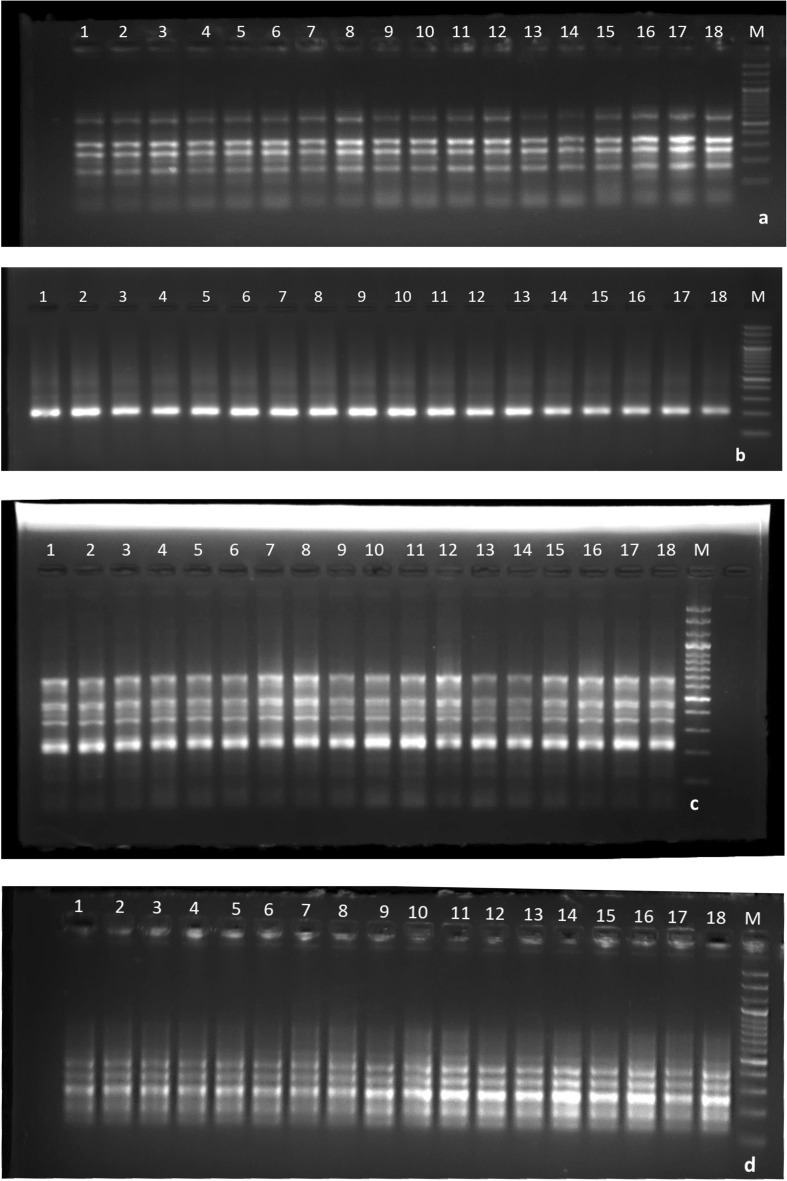
ISSR profile of primer (**a**) UBC848, (**b**) UBC858, (**c**) UBC836 and (**d**) IS53 in *Xanthosoma sagittifolium*; where 1–3: mother plants, 4–6: calli, 7–9: meristematic clumps obtained on MS + 0.2 mg/L BAP + 0.1 mg/L NAA, 10–12: meristematic clumps obtained on MS + 2.5 mg/L TDZ, 13–15: plantlets multiplied MS + 0.5 mg/L BAP + 0.05 mg/L NAA, 16–18: regenerated plantlets, M: 100 bp DNA ladder.

In the present study, different cytokinins namely BAP, TDZ, kinetin and auxins including IAA, IBA, NAA were used for shoot multiplication and root induction, respectively. Therefore, molecular profiling of the induced callus, meristematic clumps and in vitro regenerated plantlets on optimized media was done utilizing 40 ISSR primers to detect any variations with reference to mother plants. Out of 40 tested primers, 25 gave scorable amplification (62.5%). A total of 91 monomorphic bands were witnessed with 25 primers thus yielding an average of 3.64 bands per primer. A maximum number of bands (7) was obtained with primer UBC855, whereas, primers UBC858, UBC829 and UBC834 gave a single amplicon (Table 4). The size of amplicons obtained from these 25 primers ranged between 110 to 996 bp. Scoring data analysis revealed 100% similarity of all the tested samples (calli, meristematic clumps, regenerated plantlets) with mother plants that confirms the genetic uniformity of in vitro plant material during all multiplication stages on optimized media. Similar PCR amplification profiles of mother plants and in vitro multiplying samples (Fig. 6) indicates that no genetic variations are induced at the tested loci due to the use of optimized phytohormone concentrations.

**Table 4:** Detailed information and amplification profile of the ISSR primers used in the study.

S. no.	Primer code	Primer sequence (5'–3')	Annealing temperature (°C)	Total no. of bands	Band size range (bp)
	IS6	(GA)_8_C	51.2	5	110–486
	IS7	(GT)_8_A	50.7	NA	NA
	IS8	(AG)_8_C	51.2	5	199–515
	IS9	(TG)_7_ T A	54.1	2	262–920
	IS10	C(GA)_8_	49.2	Smear	Smear
	IS11	(CA)_7_G	52.5	5	166–560
	IS12	(GT)_8_C	52.5	NA	NA
	IS61	(GA)_8_ T	54.1	4	267–594
	IS53	(AG)_7_C	54.1	4	177–418
	IS65	(AG)_8_ T	52.5	Smear	Smear
	UBC859	(TG)_8_RC	51.2	4	239–996
	UBC824	(TC)_8_G	50	2	298–495
	UBC861	A(CCA)_8_CC	56.6	5	250–736
	UBC858	(TG)_8_RT	52.5	1	215
	UBC870	(TGC)_6_	51.2	Smear	Smear
	UBC825	(AC)_8_ T	52.5	3	230–424
	UBC826	(AC)_8_C	52.5	3	192–600
	UBC828	(TG)_8_A	50	Smear	Smear
	UBC829	(TG)_8_C	50	1	237
	UBC831	(AT)_8_YA	37	NA	NA
	UBC832	(AT)_8_YC	37	NA	NA
	UBC833	(AT)_8_YG	42.8	NA	NA
	UBC834	(AG)_8_YT	52.5	1	234
	UBC835	(AG)_8_YC	52.5	5	142–722
	UBC836	(AG)_8_YA	50	4	243–688
	UBC840	(GA)_8_YT	50	5	197–573
	UBC841	(GA)_8_YC	53.6	NA	NA
	UBC842	(GA)_8_YG	54.1	4	276–499
	UBC843	(CT)_8_RA	50	NA	NA
	UBC846	(CA)_8_AT	50	3	175–328
	UBC847	(CA)_8_AC	50	5	194–684
	UBC848	(CA)_8_AG	50	4	164–589
	UBC849	(GT)_8_CA	50	Smear	Smear
	UBC850	(GT)_8_CC	50	4	110–590
	UBC851	(GT)_8_CG	50	Smear	Smear
	UBC852	(TC)_8_RA	50	Smear	Smear
	UBC853	(TC)_8_AT	50	2	203–396
	UBC854	(TC)_8_AG	50	Smear	Smear
	UBC855	(AC)_8_CT	50	7	125–750
	UBC856	(AC)_8_CA	50	3	335–650

Contrasting results have been reported in a study conducted to analyze the genetic integrity of in vitro raised *Nepenthes khasiana* Hook. f. plantlets of three generations on optimized shoot/ root multiplication medium, where a progressive increase in genetic variation from 5.65 in first regeneration to 10.87% in third regeneration was reported^27^. The variation in the study might be induced due to enhanced exposure to high cytokinin (2.5 mg/L kinetin + 2.0 mg/L BAP) and high auxin (2.0 mg/L NAA) concentrations during shooting and rooting, respectively. Few other studies have also reported genetic instability due to polymorphic PCR amplification profiles in *Codonopsis lanceolate Dictyospermum ovalifolium* and *Spilanthes calva*^32–34^.

Our results are consistent with a recent report^31^ on genetic stability evaluation of in vitro raised *Ficus carica* var. Black Jack plantlets using woody plant medium containing 20 μM BAP + 8 μM IAA under different light treatments (normal fluorescent white light and four different LED spectra) that showed significant (97.87%) similarity using ISSR markers. The minor polymorphism in the report was attributed to the error dynamics of a PCR process.

## Conclusion

An efficient in vitro regeneration protocol for obtaining genetically homogeneous plantlets with high multiplication rates (43.54 shoot primordia, 13.44 shoots per culture vessel) that can be used for large scale in vitro clonal propagation of *X. sagittifolium* is reported here. With this rate of multiplication and if 10 culture vessels are used, a total of 18,490 shoot primordia and 1800 shoots can be obtained in a time span of 10 months. To the best of our knowledge, this is the first report on meristematic nodule formation, micro-morphological evaluation and genetic fidelity assessment during in vitro multiplication of *X. sagittifolium*. Regeneration pattern deciphered through histological analysis can further be exploited to enhance the rate of shoot formation. Moreover, this potentially effective protocol can be used to develop further techniques for plant improvement of Cocoyam including genetic transformation/ genome editing.

## Materials and methods

### Explant source

Phenotypically similar and healthy in vitro established cultures of *Xanthosoma sagittifolium* accessions IC0582689, IC812549, IC582686, IC582687, IC9605 and IC582690 were used as explant source. The in vitro cultures utilized for this study were already maintained on culture medium (MS + 0.5 mg/L BAP + 0.1 mg/L NAA) under the standard culture room conditions of culture room at Tissue Culture and Cryopreservation Unit (TCCU), ICAR-National Bureau of Plant Genetic Resources (NBPGR), Pusa Campus, New Delhi. Single shoots (~ 0.5 cm) excised from above mentioned cultures were sub-cultured individually on to the same culture medium in glass culture tubes (25 × 150 mm; Borosil, India) for obtaining enough mother stock cultures to conduct various experiments during the present study (supplementary Fig. 1a). All experiments were performed in accordance with relevant guidelines and regulations.

### Nutrient media and culture conditions

Nutrient culture medium comprised of basal MS (Murashige and Skoog medium) salts along with 3.0% (w/v) sucrose as a carbon source and 0.8% (w/v) agar (Hi-media, India) as a gelling agent. The culture media pH was adjusted to 5.8 prior to autoclaving at 121 °C and 1.05 kg cm^−2^ pressure for 18 min. All the cultures were incubated in culture room at 25 ± 2 °C with light intensity 40 μmol^−2^ s^−1^ for 16 h/8 h light/dark photoperiod provided by cool white, fluorescent tubes (Philips, India).

### Shoot multiplication

Shoots (0.5–1.0 cm) excised from in vitro multiplied cultures of accession IC0582689 were used as explant (supplementary Fig. 1b) for assessing the effects of different culture media combinations on in vitro shoot multiplication. A total of thirty two culture media supplemented with various concentrations of BAP, kinetin (Kn), TDZ and NAA either alone or in combination were used for the present study. Full strength basal MS medium free from plant growth regulators was used as control. The details of different culture media combinations are presented in Table 1.

### In vitro rooting

Well-developed healthy microshoots (2–3 cm) bearing 2–3 leaves were harvested carefully from culture tubes and inoculated on to half strength MS media supplemented with various concentrations of auxins including Indole-3-acetic acid (IAA), Indole-3-butyric acid (IBA) or α-naphthalene acetic acid (NAA) for inducing roots (Table 3). Half strength basal MS medium free from plant growth regulators was used as control.

### Hardening and acclimatization

In vitro regenerated plantlets possessing well developed shoot and roots were carefully taken out from the culture tubes and thoroughly washed under running tap water to remove the traces of adherent culture medium from root surfaces. The plantlets were transferred to 10 cm diameter plastic pots filled with autoclaved soilrite (Glasil Scientific Industries, New Delhi, India). The pots were moistened with ½-MS liquid medium devoid of organic supplements on every alternative day for at least 2 weeks and later regularly watered. All potted plantlets were covered with transparent polythene bags having two to three small holes for maintaining humidity. Polybags were removed after 2 weeks, and the plantlets were transferred to the soil and maintained under greenhouse conditions.

### Histological studies

Histological studies were performed on different morphogenetic structures^35^ obtained during in vitro multiplication. For this, the tissue samples were fixed in freshly prepared FAA (formalin-acetic acid–ethanol) for 24 h and stored in 70% alcohol at 4 °C. Samples were dehydrated in alcohol series (30%, 50% and & 70% v/v) followed by tert-Butyl alcohol (TBA) series (55%, 75%, 85%, 95% and 100% v/v) through definite time gap. Samples were then transferred to TBA and Paraffin oil (1:1 v/v) and left for 24 h, then shifted to TBA: Paraffin oil: paraffin wax (1:1:1) combination and kept in oven at 62 °C for 48 h followed by three changes of pure wax. Samples were embedded in paraffin and bee wax mixture. Serial sectioning was done by motorized Leica RM 2165 microtome and wax ribbons were adhered to glass slides by applying Haupt’s fluid (1 g gelatin, 2 g phenol crystals and 15 ml glycerin, 85 ml distilled water). Spreading of sections was done on hot plate at 60 °C and kept for drying at room temperature for about 2 weeks. De-paraffining of sections was done by dipping the slides overnight in xylene, and subsequently stained with safranin (1%) and fast green (1%)^36^. Prepared section slides were observed under Leica DM6 B microscope and photographs were taken using attached digital camera (Leica DFC7000 T) with the help of Leica application suite X software.

### Statistical analysis

The shoot multiplication data was recorded after 20 weeks of explant inoculation on culture media, whereas the rooting data were recorded after 12 weeks. The data is expressed as mean ± standard error (Mean ± SE). All the experiments were conducted in three replications. Arc sine transformation was performed on percent data. The effects of different treatments on various growth parameters were quantified and the significance of difference among means was determined by ANOVA by using SPSS version 16 (SPSS Inc. Chicago, USA) followed by Duncan’s Multiple Range Test (DMRT) at 5% level of significance.

### Molecular profiling to detect somaclonal variations

Inter simple sequence repeat (ISSR) markers were used to analyze the genetic stability of in vitro multiplied plantlets. For the purpose, genomic DNA was extracted from 18 in vitro samples that included 3 randomly selected mother plants (plantlets used as explant source) and three samples each from the callus, the meristematic clumps obtained on MS medium containing 0.2 mg/L BAP + 0.1 mg/L NAA and MS with 2.5 mg/L TDZ, shoots multiplied on MS + 0.5 mg/L BAP + 0.05 mg/L NAA and regenerated plantlets (shoot elongation on MS + 0.5 mg/L NAA + 0.01 mg/L BAP followed by rooting on MS having 1.0 mg/L IBA), using modified method of Moller et al.^37^.

DNA quality and quantity was assessed using NanoDrop 1000 (Thermo Fischer Scientific, USA). Forty ISSR primers were used in the study (Table 4). After optimization of PCR conditions for each primer (Table 4), individual PCR reaction [10 μL reaction volume containing 1X reaction mixture (One PCR™, GeneDireX Inc. USA), 10 μM primer and 40 ng template DNA] was carried out in a thermal cycler (Gene Pro, Hangzhou Bioer Technology Co., China) programmed to: initial denaturation at 94 °C for 5 min, followed by 37 cycles of denaturation at 94 °C (30 s), primer annealing at 37–56.7 °C (45 s) and primer extension at 72 °C (60 s) and a final extension at 72 °C for 5 min.

Amplification profiles were visualized on a Gel Documentation System (GenoSens 2100, Clinx Science Instruments Co., China) and data was recorded. The size of the PCR amplicons separated on 2.5% agarose gel was estimated using 100 bp DNA ladder.

## Supplementary Information

Supplementary Figure 1.

## Data Availability

All data generated or analysed during this study are included in this published article.
