# Supplementary material for: Histological and molecular insights in to in vitro regeneration pattern of Xanthosoma sagittifolium
Source: Sci Rep. 2023 Apr 10;13:5806. doi: 10.1038/s41598-023-33064-8 (PMC10086020; doi:10.1038/s41598-023-33064-8)
Supplement: Supplementary file 1 — Supplementary Figure 1. [file 41598_2023_33064_MOESM1_ESM.docx]

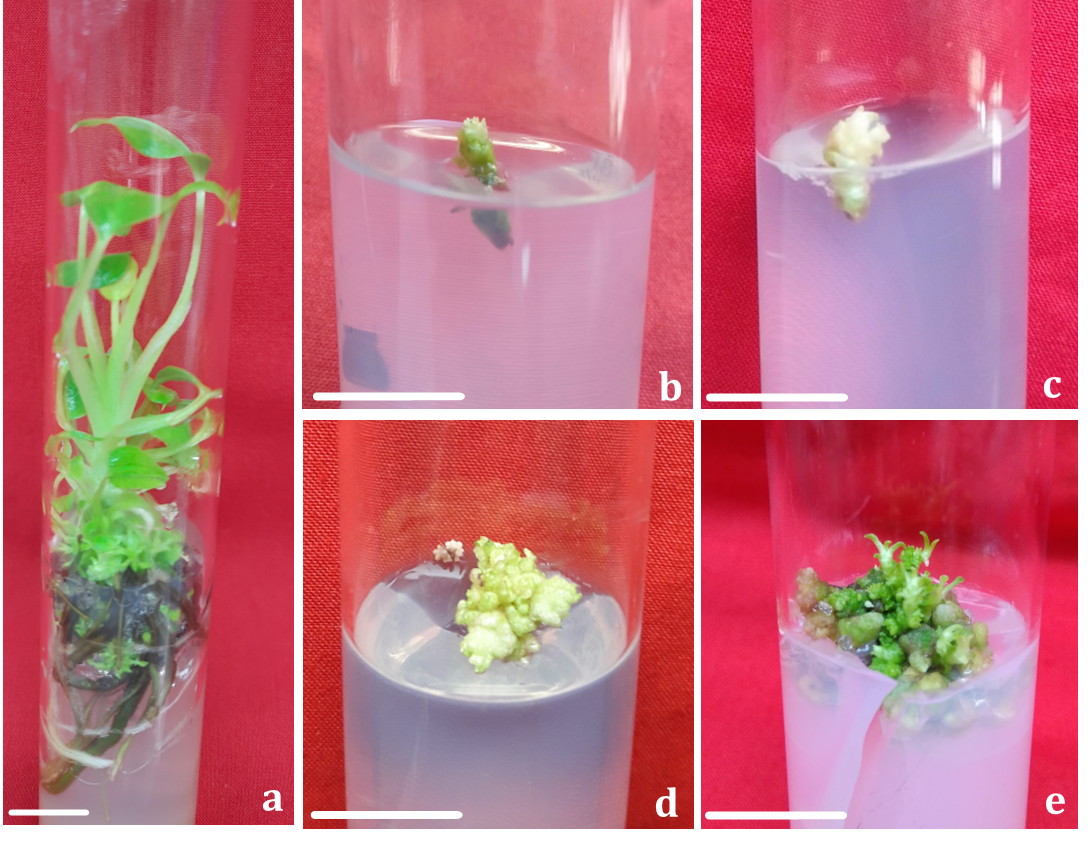


**Supplementary Figure 1:** Mother plant, stages of callus induction and shoot primordia formation (Scale bar = 1.25 cm); where a) mother plant, b) explant, c) 2 weeks old swollen explant, d) nodular callus formation, e) callus containing shoot primordia
